# Open Access to a High-Quality, Impartial, Point-of-Care Medical Summary Would Save Lives: Why Does It Not Exist?

**DOI:** 10.1371/journal.pmed.1001868

**Published:** 2015-08-25

**Authors:** James Heilman

**Affiliations:** 1 Faculty of Medicine, Department of Emergency Medicine, University of British Columbia, Vancouver, British Columbia, Canada; 2 Wikipedian at Wikipedia

## Abstract

James Heilman considers how much good could result from providing clinicians with better, freer access to the information they need to do their work.

**Summary Points**

- Currently no open access point-of-care (POC) medical summary aimed at a professional audience exists.
- Some nonprofit and multiple professional, for-profit POC medical summaries are frequently accessed by clinicians and policymakers.
- Efforts to create open access POC summaries have been stymied by the difficulty of attracting high-quality contributors.
- The open access medical publishing community can create this resource with engaged donors, crowd-sourcing, and technology.

## The Current State

Over the past decade, the world of scientific journal publishing has been transformed by open access (OA) to information. In the strict sense, open access refers to the ability for others not only to view but also to build upon and distribute a work as long as attribution of the author(s) is provided [1]. *PLOS ONE* became the world's single largest journal (by number of articles) only four years after it was founded and has since increased in volume nearly 5-fold [2,3]. Currently there are nearly 10,000 journals listed by the Directory of Open Access Journals; as of May 2015, more than 1.9 million articles [4]. About a quarter of these were related to medicine. In 2011, of all scholarly articles published, 17% were OA [5], and in the biomedical fields the proportion of freely available articles (both OA and “free” access) passed the 50% mark in 2010 [6].

The huge increase in access to scientific knowledge has been chiefly of benefit to those researchers who have the time to search the literature. It is less helpful for working health care providers, since masses of literature do not lend themselves to reliably and promptly answering questions. A recent estimate that 85% of all medical research is wasted is based on waste in the research process and in publishing the research itself [7]. This problem of waste is compounded when medical knowledge exists but health care providers have to make decisions without it, a phenomenon that has not been properly examined. One survey found that 80% of primary treatments given at a university hospital in the United Kingdom had some evidence to support their use [8]. This study, however, did not look at whether better evidence existed or how well the evidence matched the treatment decision or patients’ values, nor does it likely represent typical practices. Others have claimed that the typical rate is closer to 10% to 20% [8].

Point-of-care (POC) medical summaries exist to help physicians make decisions in ways not well served by traditional publishing formats like journal articles and textbooks. POC summaries have been described as “web-based medical compendia specifically designed to deliver pre-digested, rapidly accessible, comprehensive, and periodically updated information on a given topic to health care professionals” [9]. Unlike PubMed, Cochrane, and other resources, POC tools are designed to be quick to search and navigate and aim to help health care providers solve emerging clinical questions. As such, they are collections of “review articles” browsable in real time to aid in patient care.

## What Is Already Available?

A number of point-of-care medical summaries are available. Some are open access but not aimed at a professional audience, while those that are aimed at a professional audience are not open access. A few of the non–open access, web-based evidence summaries, while freely accessible, are funded by advertising revenue, making them vulnerable to distortion by financial interests [10]. Others, such as National Health Service’s Clinical Knowledge Summaries, are only available in Great Britain. The most popular tools aimed at professionals are subscription-only, provided by several wealthy, for-profit publishers: UpToDate and DynaMed are the most successful and best known.

Health care providers and institutions are willing to pay to access high-quality point-of-care medical summaries. Personal subscriptions for DynaMed and UpToDate are several hundred dollars for a physician and substantially more for institutional subscriptions. UpToDate company data states that its entries are the result of over 6,000 authors, editors, and peer reviewers. It reports that it is used by over “1 million clinicians in 174 countries and [that] almost 90% of academic medical centers in the United States rely on UpToDate to provide the best care.” The word “rely” is a curious one, but usage statistics support the idea that health care providers find UpToDate helpful, with more than 23 million topic views logged each month [11]. The company advertises research showing that its use is associated with “…reduced length of stay, lower risk-adjusted mortality rates, and better quality performance” [12]. Based on visits, UpToDate was within the top 50 health sites on the Internet in March of 2015 [13].

While the value of these tools cannot be assessed by observation or anecdote alone, it is clear that clinicians seek out, use, and personally pay for them. The wider world is also interested in health care content. Google states that one in twenty searches is for health care information [14]. Additionally, Wikipedia’s popularity among both physicians and the public is a further indication of global demand [15].

## What Hasn’t Succeeded?

Given the rise of OA scientific publishing, why have we not seen similar developments making the world’s medical knowledge freely available in a constantly updated and intuitively accessible format ready at the point of care? There have been attempts. But they serve not only as examples that such databases are desired but also that creating them is difficult.

In 2009, Medpedia was formed with venture capital, and their website stated they had the support of top universities, including Harvard Medical School, Stanford School of Medicine, and the University of California Berkeley School of Public Health [16]. Medpedia never caught on, and with few readers or contributors, it ceased in 2013. In 2011, WikEmerg.ca was created. It initially paid physicians to write content, but experienced trouble getting engaged contributors even when money was attached. Efforts to create broader professional encyclopedias have also failed. One of the first was Nupedia in 2000, which grew slowly under Jimmy Wales and Larry Sanger. Wales and Sanger decided to launch a website with looser contribution requirements and, in 2001, created Wikipedia. Nupedia faded, but Sanger gave the model a second try in 2007 with the launch of Citizendium. As of September 2014, it had less than 160 high-quality articles and struggled to raise enough money to stay online [17]. Even Google has tried and failed. In 2008, they started a supposed “Wikipedia killer” called Google Knol [18]. The site closed in 2012. Google Knol’s failure has been attributed to several factors, including low numbers of contributors, poor reward mechanisms, and low potential for collaboration. It rapidly filled with plagiarism and poor-quality content [19].

## Wikipedia

In medicine, the one web-based, crowdsourced project that seems to have had a degree of success is Wikipedia [15]. Ironically, the platform is geared towards the general population and not health care providers. Furthermore, Wikipedia does not intend readers to use the site to treat themselves or others. The quality of Wikipedia entries is a topic of vigorous debate, and even with its semiformal internal peer review process, the small size of the active medical community means fewer than 1% of medical articles have passed review. Yet medical information on Wikipedia is widely accessed. About 5 billion page views were logged for its medical content in 2013. Of medical students, 92% to 94% use it, and 50% to 70% of practicing physicians report also doing so [15,20,21]. A 2009 survey of UK junior physicians found that 70% use Wikipedia on a weekly basis, making it the most used content provider, ahead of eMedicine.com, which was used by nearly 50% [22]. These numbers, as with those for UpToDate, suggest a widespread desire and need for point-of-care clinical information.

## Creating an Open Access Point-of-Care Summary

### Content

An open access professional database would need open access professional content. Such a database could be created “de novo,” it could be developed from currently available open content, or it could be constructed by releasing what is currently closed-source content under open licenses. Even if a benefactor with deep pockets were willing to buy UpToDate or DynaMed and make its content freely available as a starting point, mechanisms to regularly update the content remain essential.

Creating entirely new content is an enormous undertaking, but with the support of OA publishing and the wider medical community, it may be possible. UpToDate has around 10,000 topics [9], while eMedicine is estimated to contain 7,000 [23]. Likewise, Wikipedia contains about 7,000 disease- or symptom-related articles. As previously mentioned, there are about 2,500 medical OA publishers. If each was to commission a single broad-topic review each year, they would quickly match the scope of content contained within these commercial sites.

There is a great deal of existing open access content that could be adapted to be more professional and clinically useful. This includes content from Wikipedia, the US Centers for Disease Control and Prevention, the Food and Drug Administration, and the National Institutes of Health (NIH). The NIH, for example, has open access content spread across at least 27 different institutional websites. Even finding the correct website is, therefore, a challenge when searching for the topic one is looking for. Additional material could come from other organizations if they could be convinced to release content more openly; these might include the World Health Organization, National Health Services in the United Kingdom, and Health Canada. The Bulletin of the World Health Organization began using an open license in July of 2014. Medication-related information could possibly come from organizations such as the American Society of Health-System Pharmacists or the Royal Pharmaceutical Society, which already produce the American Society of Health-System Pharmacists Drug Information and British National Formulary, respectively.

A team of editors would then give the final product a consistent style, find appropriate peer review, and verify quality. How, though, should this team and the website by which the information was delivered be paid for?

### Funding Model

Expenses for a medical project such as this relate not just to platform development but also content creation, editing, production, and promotion. Recruiting quality content has proved a stumbling block for some previous efforts despite offering payment for written work. Unpaid content creation draws upon the established practice of many scientific journals; editors commonly expect experts to write review articles for free, with the joint rewards of increased visibility for their ideas and a new publication on their professional record. For an open access POC medical summary to attract quality content, it must likewise confer prestige or career advancement to its authors. Most open access journals, including *PLOS Medicine*, do not contain literature-review–type articles (not to be confused with systematic reviews). Expecting authors to pay to write articles, as in a current open model for primary research, would not be realistic for reviews unless publication, as with research, also lead to career advancement.

Wikipedia has a surprisingly small community of contributors, perhaps partly because it lacks prestige among the academic community. If contributing to a global knowledge database came to be valued, in terms of prestige and career progression, contributors would compete for the right to help. Once the reputation of such a database became established, it would be self-reinforcing, encouraging ever more competition to contribute, with resulting increases in quality and prestige for those involved. There would also be something honourable about spending part of one's career on such an effort.

Wikipedia’s funding model relies almost entirely on donations from readers. This is relatively effective, even given the small percentage who contribute, as nearly half a billion individuals use the site each month. About 2 million people donated an average of US$15, mostly during a one-month period last year. Still, Wikipedia runs on a low budget of less than US$50 million a year, as compared to annual revenues of US$4.7 billion for the parent company of UpToDate [11] and US$2 billion for the parent company of DynaMed [23]. The Wikimedia Foundation, the non-governmental organization (NGO) behind Wikipedia, developed slowly, and it is only in the last few years that they surpassed 100 employees and US$10 million [24]. To seriously undertake the establishment of a global clinical knowledge database, of sufficient quality to become self-sustaining through acquiring status and accepted utility, would require inputs of tens of millions of dollars a year. Arguably it might also be an incredibly cost-effective way of improving global health, simply by making sure the best medical knowledge was freely available to those who needed it.

### Copyright

The NIH currently pays for licenses to use some material for their medical encyclopedias. Even though hosted on a .gov website, it is not in the public domain or under an open license. The content is basic, containing no inline references, and is too simple to be useful to a professional.

The World Health Organization (WHO) and National Health Services (NHS) have much high-quality material that can be freely viewed online, but very little of it is under an open license. The WHO material, additionally is often not in a format that is easily viewed, with much of their content in large, image-laden PDFs that are slow to load even for those with excellent internet connections. While the WHO’s policy on open access is now supporting greater availability of the material it produces, it has not yet gone so far as to make all of its content available under an open access license, such that others can build upon it. Additionally the organization occasionally uses the term “open access” when they appear to mean “free access” [25]. One would hope that it would be possible to persuade these organizations to embrace true open access on the grounds that this release of knowledge would be consistent with their aims of advancing health.

The US federal government has been releasing much of what they produce into the public domain since 1895 [26]. The benefits to science and health of this position have been much greater than any harms. This is the reason, for example, we have access to the complete human genome. There is little evidence that others have taken their work, altered it, and claimed that it is still the position of the US government. United Nations Educational, Scientific, and Cultural Organization (UNESCO) began using open access licenses in July of 2013, as they see this as “key to the advancement of innovative solutions for the challenges of international development” [27]. This applies to all content published after July 2013, with some older content also being relicensed.

Problematic among those who do use open licenses is that there is not just one creative commons (CC) or open license, but many exist and they are often incompatible or only compatible in one direction. Qualifiers of open licenses include “by attribution” (BY), “share-alike” (SA), “non-commercial” (NC), and “no-derivatives” (ND). Many NGOs create content under CC BY SA NC licenses that are not usable within a CC BY or CC BY SA source. If such acronyms seem confusingly bad reasons for not sharing knowledge that could improve global health, so they should. Efforts at Wikipedia to improve reliability included efforts to work more closely with PLOS and BMJ Open, but these efforts faced copyright issues. A common license for open access journals is CC BY, while Wikipedia’s content is under an incompatible CC BY SA. Thus, Wikipedia content cannot be published by these journals under their usual license. Neither PLOS, BMJ Open, nor Wikipedia were able to find a way to align their licenses. These licensing issues make it difficult for those who are working towards the same goal of “health information for all” to collaborate. The NC license faces the additional issue that what defines a non-commercial use is not clear. For example, while many view education as non-commercial, students pay to attend university, and professors make a living teaching and doing research.

### Global Access

Getting access is key. Simply putting content up on the Internet is not enough—many people, including many health care providers in low- and middle-income countries, do not have easy or inexpensive access to the Internet. Most people, however, do have access to cell phones. The Wikimedia Foundation has agreements with cell phone companies to provide Wikipedia without data charges in low- and middle-income countries. As of May 2015, 400 million people in low- and middle-income countries have free access to Wikipedia without data charges through these programs. This suggests a model for widely disseminating a clinical knowledge database. Loading health content on phone systems before they are shipped may be another option and is one Wikipedia is currently pursuing.

## Looking Forward

The success of PubMed Central and the many open access journals in making medical knowledge openly available to researchers has been immense. Nothing similar has yet been done for clinicians. Our immediate goal should be to provide accessible, high-quality medical information that is easily searchable and usable at the point of care (Box 1). Using for-profit POC medical summaries as a yardstick, this would involve the putting together of 5,000 to 10,000 overview articles. To have global impact, this content would need to be available in languages comprehensible by the majority of the world’s health care providers. It would need to be updated on a regular basis and available in multiple formats, including on the web, as e-books, and in spoken form. It would also need to be freely readable, including without data charges in the developing world.

**Box 1. Options**

**Costs low, risk minimal, control limited**

Established medical publishers work to engage clinicians, academics, and government agencies in an effort to generate high-quality, open access POC summaries. Open access summaries could initially be developed via commissions from high-impact journals. Once a database of summaries is established, prestige will reinforce content contribution. Experts can follow their conscientious desire to promote global health while benefiting professionally.

Open access journals would need to agree on a consistent style and compatible license. Funders of research such as the NIH and NHS could put money towards these efforts and benefit from reuse of the materials on their own websites.

This is already happening to a limited extent in a slightly different format, with the first Wikipedia article formally published following peer review in the journal *Open Medicine* in October of 2014 [28]. Cancer Research UK and ECGWiki have released large number of images under a more open license and organizations including the Cochrane Collaboration, UCSF School of Medicine, Cancer Research UK and the National Institutes of Health are working to improve Wikipedia content.

**Costs moderate, risk moderate, control moderate**

A medium-sized not-for-profit could be set up and funded through grants, donations, and goodwill. Membership would come with expectation of openness. This would hopefully push some organizations to reconsider their position and become more serious about the importance of open licensing.

Create and manage a MediaWiki website editable only by approved users. Build up a collaboration of partners. Develop a manual of style and outlines. Petition partners to fill in portions with stipends and authorship as rewards. Use both internal and external peer review. Encourage content from Wikipedia and inclusion into Wikipedia to expand audience. Hire programmers to manage automated scripts and develop the underlying software to better fit the needs of the site.

**Costs significant, risk large, control greater**

Build from the ground up, create a new website and software, hire authors, hire translators, convince cell phone companies to allow free use, and raise capital. Would require convincing major funders to invest large amount yearly and in perpetuity. Would require hundreds of employees to manage all aspect of a large publishing and distribution house.

Such a database is not a small goal, but it is a worthy one. That medical decisions are made without timely access to the existing evidence base, and that this occurs in a world which recognises the importance of open access to scientific knowledge, should not be allowed to continue. An opportunity exists for governments, NGOs, IGOs, and philanthropists to reduce the global burden of disease simply by working together to make existing medical knowledge easily, freely, and usefully available.

## Abbreviations:

BY: by attribution
CC: creative commons
NC: non-commercial
ND: no-derivatives
NGO: non-governmental organization
OA: open access
POC: point of care
SA: share-alike
